# The Potential Link between Thermal Resistance and Virulence in *Salmonella*: A Review

**DOI:** 10.3389/fvets.2017.00093

**Published:** 2017-06-14

**Authors:** Turki M. Dawoud, Morgan L. Davis, Si Hong Park, Sun Ae Kim, Young Min Kwon, Nathan Jarvis, Corliss A. O’Bryan, Zhaohao Shi, Philip G. Crandall, Steven C. Ricke

**Affiliations:** ^1^Cell and Molecular Biology Program, University of Arkansas, Fayetteville, AR, United States; ^2^Center for Food Safety, University of Arkansas, Fayetteville, AR, United States; ^3^Department of Food Science, University of Arkansas, Fayetteville, AR, United States; ^4^Department of Poultry Science, University of Arkansas, Fayetteville, AR, United States

**Keywords:** *Salmonella*, thermal stress response, heat shock proteins, sigma factor, virulence

## Abstract

In some animals, the typical body temperature can be higher than humans, for example, 42°C in poultry and 40°C in rabbits which can be a potential thermal stress challenge for pathogens. Even in animals with lower body temperatures, when infection occurs, the immune system may increase body temperature to reduce the chance of survival for pathogens. However, some pathogens can still easily overcome higher body temperatures and/or rise in body temperatures through expression of stress response mechanisms. *Salmonella* is the causative agent of one of the most prevalent foodborne illnesses, salmonellosis, and can readily survive over a wide range of temperatures due to the efficient expression of the heat (thermal) stress response. Therefore, thermal resistance mechanisms can provide cross protection against other stresses including the non-specific host defenses found within the human body thus increasing pathogenic potential. Understanding the molecular mechanisms associated with thermal responses in *Salmonella* is crucial in designing and developing more effective or new treatments for reducing and eliminating infection caused by *Salmonella* that have survived heat stress. In this review, *Salmonella* thermal resistance is assessed followed by an overview of the thermal stress responses with a focus on gene regulation by sigma factors, heat shock proteins, along with the corresponding thermosensors and their association with virulence expression including a focus on a potential link between heat resistance and potential for infection.

## Introduction

*Salmonella* is a Gram-negative foodborne pathogen that is a major concern for the food industry and public health authorities because of its capability to cause both widespread contamination and infection within the United States (US) and worldwide (1–5). An estimated one million cases of *Salmonella*-related illnesses occur annually within the US. For example, in 2014, *Salmonella* was responsible for 10 multistate outbreaks with approximately 1,000 reported outbreak cases (3, 6). Numerous strategies have been implemented to reduce *Salmonella* transmission, contamination, and infection. *Salmonella* infections are most commonly acquired through ingestion of contaminated foods such as eggs and poultry meat (7). *Salmonella* can colonize the small intestines of poultry birds, along with the cecum, without demonstrating any symptoms related to *Salmonella* infections. Therefore, poultry serves as an efficient vector of transmission for multiple serovars of *Salmonella* to humans through consumption of contaminated food products.

In order for *Salmonella* to survive and colonize the human body, it must overcome multiple non-specific host defenses encountered within the host such as low pH, limited nutrient availability and in poultry birds, a high body temperature (42°C). Due to the wide temperature range that *Salmonella* may grow in, it must possess specific mechanisms that can overcome thermal stress to proliferate and survive. However, prior to ingestion, *Salmonella* is already preexposed to a higher core body temperature in poultry compared to humans (37°C). During infection, one of the primary defenses of the innate immunity is an increase in body temperature through pyrogens (antigens that stimulate fever) such as lipopolysaccharide found in the cell wall of Gram-negative bacteria (8). This preexposure could increase the potential of *Salmonella* to establish infection of the host due to adaption to higher temperatures. Therefore, the aim of this review is to provide an overview of phenotypic and molecular responses to temperature changes as it relates to poultry, thermal stress regulation, and how this increases pathogenic potential of *Salmonella*.

## Thermal and Non-Thermal Stresses

With over 2,500 serovars of *Salmonella*, several have developed the ability to overcome high temperatures allowing for survival through thermal processing; however, this is strain specific (9–11). O’Bryan et al. (12) reviewed the thermal resistance of *Salmonella* species and other foodborne pathogens associated with meat and poultry. They concluded that a variety of factors and parameters are involved in the thermal resistance and inactivation of those pathogens and spoilage microorganisms such as various temperature exposures, growth phase, and the intrinsic conditions of the food product. Strains of the same microbial species were found to be capable of responding differently to the same treatments possibly due to specific variations in gene composition for each respective strain. Likewise, the stages of growth, the age of the culture, and the conditions of bacterial growth have yielded various outcomes regarding heat inactivation or destruction of microorganisms, which could contribute to determining the best methods to reduce microbial growth and contamination within these products (12).

There are several factors that allow *Salmonella* strains to survive the food processing environment and overcome thermal treatment. For example, preexposure to stress and growth conditions prior to thermal treatment could increase survival capability during processing. Specifically, *S*. Senftenberg was found to survive in broiler litter for up to 24 h at 80°C (13). Microorganisms tested against heat are known to elicit different responses in regard to prior growth conditions with stationary phase cells being more resistant to heat than log phase cells (14–16). In addition, stressed cells such as those exposed to temperatures slightly above an organism’s optimal growth range (heat shocked cells), those grown on limited carbon sources, those experiencing desiccation, and those undergoing starvation stress prior to heat treatments have been shown to exhibit more thermal tolerance (17–21).

Exposure to non-thermal stress may also have an impact on the capability of *Salmonella* to respond to thermal threats. For example, Milillo and colleagues concluded that combining organic acids with heat can effectively reduce *Salmonella* over a short period of time (22, 23). They applied mild thermal treatments and organic acids with a 1-min exposure time. Sodium propionate in combination with heating was demonstrated to be the most significantly effective for reducing viable *Salmonella* (22). In a follow-up study, Milillo et al. (23) conducted microarray experiments to explore the specific response of *S*. Typhimurium to organic acids in combination with heat. Exposure to sodium acetate with heat (55°C) and sodium propionate with heat (55°C) led to differentially 288 (124 upregulated and 168 downregulated genes) and 319 (131 upregulated and 181 downregulated genes) gene expression level changes, respectively. Numerous heat shock genes including *dnaK, hptJ, dnaJ, grpE, clpP*, and *hscAB* were repressed by both treatments. They concluded that this synergism may be attributed to damage in the synthesis of heat shock genes of *S*. Typhimurium due to membrane damage. Given the potential for such synergism among otherwise unrelated interventions, there may be opportunities for optimizing hurdle technologies in the food industry and demonstrating the utility of using genomic screening to develop application approaches for these technologies.

## Thermosensors

In order for *Salmonella* to overcome and adapt to an ever-changing environment it must overcome stressors encountered during its travel through the host; therefore, adaptation through sensory mechanisms is imperative. Thermosensors are considered the cell’s “thermometer” by utilizing various types of biological systems to detect temperature fluctuations within the cell. There are four different groups of thermosensors including proteins, lipids and membrane fluidity, RNA’s that are temperature responsive, and DNA structure and topology. Thermosensors play a major role in temperature detection and are found within the 5′ UTR region, which can regulate gene expression to produce adaptive heat stress responses. When temperature decreases or increases to harmful levels, stress responses (cold and heat shock) are needed to protect the bacterial cell and are thoroughly dependent on bacterial signal transduction mechanisms (24). Genes involved in these mechanisms are regulated at different genetic stages beginning from transcription through translation and into posttranslational levels (25, 26).

As a protective reaction, misfolded and unfolded proteins are present in considerable numbers in the cytoplasmic membrane and the outer membrane during exposure to higher than optimal temperatures which, in turn, initiates the expression of heat shock proteins (HSPs) through the regulation of the heat shock factor σ^H^ (27–31). Proteins involved in heat shock are summarized in Table 1. Induction of HSP formation is accomplished through the production of chaperones, proteases, and small heat shock proteins (s-HSPs). These function in protection, refolding salvaged proteins, removing damaged proteins, and repairing degrading protein aggregation (32–37).

**Table 1:** Proteins involved in heat shock and their function are described.

Summary of proteins involved in heat shock		
Protein	Function	Reference
DnaK	DNA replication under heat shock; chaperone protein	(23)
DnaJ	Prevents aggregation of denatured proteins under hyperosmotic and heat shock	(23)
GrpE	Nucleotide exchange factor for DnaK; thermosensor	(23)
ClpP	Protease that degrades regulatory proteins	(23)
HscAB	Chaperone; maturation of iron–sulfur clusters during heat shock	(23)
σ^H^ and σ32	Regulators heat shock response; controls envelope stress response to heat shock, acid stress	(27, 38–40)
FourU	Thermosensor; temperature-responsive RNA element	(40–46)
TlpA	Unknown but suggested to be a transcriptional regulator	(25, 47)
HtrA	Thermosensor endopeptidase; chaperone in the outer membrane and degrades misfolded proteins	(48–54)
RpoS^a^	General stress response sigma factor; DNA repair under stress	(55, 56)
FkpA	Involved in intracellular survival of macrophages	(57, 58)
SurA^a^	Outer membrane protein development and assembly; folding of proteins involved in transportation channels	(59–64)
H-NS	Virulence factor regulator under thermal changes	(65–70)

*^a^Those involved in both heat shock and virulence*.

FourU is a family of thermosensors located at the untranslated region (5′-UTR). This temperature-responsive RNA element contains a stretch of four uridine nucleotides within the ribosomal binding site. It pairs with a sequence of AGGA and was initially discovered in *S*. Typhimurium as the small heat shock gene *agsA*, aggregation suppression A (40–46). Afterward, a similar RNA thermometer was also confirmed to be associated with *Yersinia* virulence through the induction of the transcriptional activator *lcrF* (44, 71).

TlpA, TIR-like protein A, is considered one of the first reported proteins with thermosensory gene regulation activity to the high temperature response (HTR) encoded by *Salmonella* enteric virulence plasmid, pSLT (25, 47). It is a robust homolog to a eukaryotic protein family known as tropomyosin, and the structure of TlpA is in a dimer form with an unusually long alpha-helical coiled coil structure (72). It consists of an N-terminal DNA binding domain and exhibits transcriptional autoregulatory repression activity. At temperatures below 30°C, the transcription of *tlpA* is low and the TlpA repression activity is high. The TlpA exists in two forms, as a dimeric α-helical (folded) coiled coil oligomer at low temperature (28°C) and an unfolded (non-functional) monomer at high temperature (37°C) that leads to increased transcription (25, 73, 74). Although the function of this protein is still unidentified, it was demonstrated that this transcriptional regulator was not essential for virulence of *Salmonella* using a mouse infection model (75).

Another thermosensing gene known as *htrA*, high temperature requirement A, is a member of the serine proteases group within the endoproteases family and is regulated by sigma factor E (48–52). It is a highly conserved gene in numerous microorganisms and was first discovered in *Escherichia coli* as *degP*. At low temperature, the protein HtrA (DegP) functions as a chaperone in the outer membrane; however, at high temperatures, it acts as a protease to degrade misfolded proteins with ATP-independent activity and other cofactors (53, 54). An earlier study also linked the activity of this gene to its sensitivity to thermal stress (76). A strain with a mutation in this gene exhibited an inability to grow at high temperature characterized by the inability to degrade unfolded proteins in the periplasmic space. *S*. Typhimurium was less affected by the sigma factor E mutation than *E. coli* (77–79).

## Cellular Responses and Regulation to Heat Stress

*Salmonella* can proliferate either in a planktonic form, floating freely within a liquid medium, or attach and grow while immobilized to a solid medium. A large number of proteins form the family of s-HSP that consists of proteins with up to 50 amino acids, which are considered energy free and universally found in numerous microorganisms with diverse group and variable molecular weights. These proteins possess chaperone-like functions and commonly maintain protein homeostasis. The s-HSPs are active primarily during stress to stabilize cell proteins at diverse cellular activities (metabolism, translation, transcription, and others), binding unfolded proteins and forming a complex that blocks non-specific irreversible aggregation (80–83). With the detection of heat stress, the adaptive regulation of genes is initiated with the expression of sigma factors. Two sigma factors are generally expressed: a cytoplasmic thermal stress response regulated by heat shock sigma factor, σ^H^ or σ^32^, and an extracytoplasmic thermal stress response regulated by the extracytoplasmic function sigma factor, σ^E^ or σ^24^, also known as extreme heat stress sigma factor (84–88).

Sigma factors comprise a large group of genes expressing proteins with critical mechanisms associated with the RNA polymerase holoenzyme complex that function as guidance for core RNA polymerases to recognize their promoters and initiate transcription. The sigma factors are primarily divided into two categories, sigma factor 70 family (σ^70^) that coordinates the transcriptional activities in various stress responses, also known as σ^A^ in *Bacillus subtilis* and other bacterial species (51, 89–91), and a second identified family of sigma factors encoded by *rpo^N^*, known as sigma factor 54 (σ^54/N^) (92–94), identified in *Campylobacter jejuni, Enterococcus faecalis, Listeria monocytogenes*, and *Pseudomonas* spp. (29, 95–97).

Heat shock responses are regulated by the alternative sigma factors σ^32/H^ and σ^24/E^. These two factors make up the third and fourth subgroups of sigma factors encoded by *rpoH* and *rpoE* genes, respectively (98, 99). *rpoH* regulates the transcription of heat shock genes and is itself regulated during translation. When the temperature is at an optimal microbial growth range, the translation of the *rpoH* gene is blocked. The stem III and I of the *rpoH* mRNA secondary structure is liberated with increasing temperatures (42°C), facilitating the ribosomal binding and enhancing the efficiency of translation (27, 38–40). Sigma factors associated with heat stress response have been demonstrated to regulate over a 100 genes. Of those, sigma factor σ^32/H^ controls more than 30 proteins, most of which are associated chaperones and proteases (30, 31, 100–103). A more recent study by Lim et al. (104) made it clear that σ^32/H^ is not just localized at the bacterial cytoplasm but is also found in the inner membrane through a direct interaction with the signal recognition particle and its signal receptor.

Proteases expressed by sigma factor σ^32/H^ can control and decrease the expression of the membrane HSPs to a level as needed by the cell to withstand environmental stresses. For instance, FtsH is one of the ATP-dependent proteases, which possesses numerous cellular functions and has been demonstrated to be very critical to *E*. *coli* viability (105–108). In addition, FtsH functions as a protein qualifying protease and has a role in membrane protein degradation activities primarily those with SsrA-tagged cytoplasmic proteins at their carboxy terminal (109, 110). FtsH degrades MgtC, a membranous protein with five transmembrane domains. MgtC, a virulence factor, has been identified as being required for survival inside macrophages (111). Katz and Ron (108) demonstrated a maintenance role of FtsH for lipopolysaccharide biosynthesis with a shielding permeability function (108, 112–114). Although σ^32/H^ and σ^24/E^ are alternative sigma factors, σ^32/H^ regulates HSPs for the cytoplasmic components and σ^24/E^ regulates the extracytoplasmic (cell envelope) proteins in response to high temperatures and other envelope stress factors (50, 52, 88, 115–118). An interesting finding is that one of the four promoters of *rpoH* gene expression is regulated by σ^24/E^ for additional coordination of thermal responses requiring both cytoplasmic and extracytoplasmic components (119–124).

## Heat Shock and Virulence

The adaptation of *Salmonella* to heat shock can also lead to a range of other effects, including an increase in virulence potential through gene regulatory mechanisms. Exposing *Salmonella* to thermal stress results in protective responses and can induce changes in gene expression levels of virulence genes. Numerous chaperones and proteases regulated by the alternative heat shock factors, σ^H/32^, σ^E/24^, and others such as σ^S^ (RpoS) are notably involved in bacterial virulence with several studies linking these proteins to *Salmonella* and *E*. *coli* virulence factors (51, 125–129). Although both σ^H/32^ and σ^E/24^ are regulators for heat shock stress, their molecular mechanisms for initiation responses are not similar.

Sirsat et al. (130) examined the effect of heat stress on *S*. Typhimurium gene expression using transcriptional profiling. Microarray analysis was applied to identify the thermal stress response of *S*. Typhimurium at a sublethal temperature of 42°C with 144 upregulated and 167 downregulated genes detected. These genes belonged to various functional categories, but primarily to the general stress response sigma factor S (RpoS) and HSPs, and to sigma factors H and E (RpoH and RpoE). The latter protein has been shown to be critical in the virulence of numerous pathogens (131–133). However, RpoS regulates genes responsible for lethality in mice where preadaptation through RpoS by increasing virulence potential of *Salmonella* cells that survive processing as suggested by Dodd and Aldsworth [(55); Ibanez-Ruiz et al. (56)]. Therefore, sigma factors and HSPs may increase pathogenic potential by overcoming various stressors and increasing pathogenic and colonization potential. Interestingly, research has indicated that RpoS can function as a DNA repair protein that is active under stressful conditions. Thermal stress can induce DNA damage suggesting that there is correlation between thermal stress, the general stress response, and virulence of *Salmonella* (56). However, more research is needed to confirm this. Generally, genes associated with stress and energy metabolism represent the first responses of the cells to tolerate heat stress. These genes may possibly give the pathogen cross-resistance to other stresses and result in more virulent cells. The study conducted by Sirsat et al. (130) was considered the first to report that sublethal heat stress-influenced *Salmonella* interaction with Caco-2 cells through the expression of fimbriae-associated genes. Genes of two *Salmonella* pathogenicity islands (SPI-2 and SPI-5) were upregulated, resulting in improved adhesion (SPI-5 only) and survival in the host while genes of SPI-1 were downregulated.

A loss of *rpoE* gene activity has also been shown to cause a defect in cell viability of *E. coli* and increase cell envelope stress (50, 118, 122, 134). In *Salmonella, rpoE* mutants were found to be less responsive to heat shock temperatures, exhibiting an intracellular defect in the survivability within a macrophage and becoming avirulent in a mouse infection model (126, 135–137). In addition, the *rpoE* gene has been shown to be essential in response to starvation stress (138), oxidative stress (92), antimicrobial peptide resistance (139), and osmotic and cold stresses (127). Lewis et al. (128) discovered that both functions of *htrA*, are important with the function of the proteases being most critical inside the host. A more recent study verified that HtrA protein activity is critical for *S*. Enteritidis persistence in egg whites at 42°C (129).

FkpA, an FKBP-type periplasmic peptidyl-prolyl cis/trans isomerase (PPIase), is involved in heat tolerance (116). This protein is comparable to proteins known as macrophage infectivity potentiators found in other pathogenic bacteria and improves the survivability and proliferation inside the macrophages and epithelial cells (140). Horne et al. (141) demonstrated that a mutation in *fkpA* causes the corresponding *Salmonella* strain to become avirulent; however, Humphreys et al. (57) argued that a single mutant deletion of *fkpA* was not enough to reach that conclusion. They observed that only when combining that mutation with one of the other σ^E^ regulated genes, *surA* or *htrA*, would the virulence of *S*. Typhimurium be disrupted (57, 58). In a more recent study reported by Weski and Ehrmann (142), they conducted a genetic analysis of chaperones and proteases of *E. coli* associated with the cell envelope, evaluating single and double mutant deletions under different growth conditions. A *fkpA* mutation was examined at 37 and 42°C using rich medium agar plates with and without 0.5 M NaCl with the corresponding mutants found to not exhibit any detectable defects under any of the conditions. However, when combining this strain with another mutation in *dsbA*, disulfide bond formation A, the strain displayed weak growth on the hyperosmolar media when incubated at 37°C, while no sign of growth was observed on the hyperosmolar media when incubated at 42°C with a minimal growth of *dsbA* single mutants at the latter condition (142, 143).

SurA, survival protein A, is also a PPIase. It is regulated by σ^E^ and contributes to thermotolerance fitness. This protein participates in outer membrane protein (OMPs) development and assembly and plays a role in the folding of transportation channels, known as porins (59–64). Sklar et al. (62) observed that the *surA* role is associated with the initial phases of OMP biosynthesis. Previously, Tormo et al. (144) had demonstrated that *surA* was critical to *E. coli* for survival during stationary phase. Tamae et al. (145) screened approximately 4,000 single mutant deletions, among them a Δ*surA* that exhibited chemical sensitivity to the drugs and detergents used in the study. It is not clear whether similar functionalities exist with *surA* in *Salmonella* but it does appear to have the same association with virulence in the host. Sydenham et al. (146) found a mutation of *surA* in *S*. Typhimurium that exhibited extensive attenuation when introduced to mice orally or intravenously. It has also been demonstrated in several studies that *surA* is a critical factor for OMP transport and associated with virulence of uro-pathogenic *E*. *coli* and *Salmonella* (64, 147). Using a high-throughput Tn-seq technique to screen the entire genome, Khatiwara et al. (148) identified numerous genes in *S*. Typhimurium associated with high temperatures with *surA* identified as a gene associated with growth at 42°C.

Numerous studies have associated virulence factors with thermal changes that mediate DNA topology. These modifications include overall DNA helical conformation “supercoiling,” the degree of helical twists and coiling (25, 149–152), or alterations in the specific-sequence curvature of chromosomal or plasmid DNA (153–156). Some studies have demonstrated that DNA topology plays a role in *Salmonella* pathogenicity (157, 158). Positive DNA supercoiling after heat exposure causes DNA to be twisted in a right-handed fashion until it generates a knot, as seen mainly in plasmid DNA, and is controlled by DNA gyrase and topoisomerase I. Changes at the level of DNA supercoiling trigger SPI-1 gene expression levels and initiate the subsequent intestinal invasion. Once inside the host cells, the DNA changes its form and as a result, SPI-2 genes are induced (159, 160). For a more detailed discussion of SPI-1 and SPI-2 regulation, please see Ref. (160).

The second mechanism is through a recognized bending DNA sequence “promoter-curvature.” Commonly, this bending DNA region is an AT-rich sequence that has been primarily identified in the 5′-end upstream of the promoter region influencing RNA polymerase binding as a silencing factor. Initially, thermal stress induces some alterations in the DNA topology as bends in the AT-rich sequence regions on the transcriptional level. This can influence the interaction between RNA polymerase and the promoter region, altering gene expression (155, 156).

## Cross Protection

The microorganisms’ responses to temperature changes (inflammation, fever) vary from one microorganism to another with cell metabolic changes occurring when sensing external environmental shifts resulting in protection from certain stresses and/or cross protection for other additional stresses (130, 161, 162). This can be a major concern within the host by increasing potential for overall pathogenesis. Prior exposure to prevention strategies utilized within industry before human consumption occurs could increase survivability of *Salmonella* and their ability to establish infection once ingested (163). When Nielsen et al. (164) compared two different growth forms of *S*. Typhimurium, immobilized versus planktonic cells, diverse responses were elicited in response to heat shock at 45°C for 30 min. The results revealed that 538 genes were expressed differently with flagellar and virulence genes upregulated in the immobilized heat stressed cells compared to the non-stressed cultures. Greater invasiveness was observed in immobilized HeLa cells after this sublethal treatment compared to decreased invasiveness in the planktonic cells. Based on this study, it would appear that inadequate cooking and heat treatments during food processing could actually increase survival and thermal resistance of *Salmonella* and other foodborne pathogens through cross protection by increasing virulence capability (164–166). Gruzdev et al. (21) found that desiccated *Salmonella* cells in sterile deionized water showed high tolerance to dry heat at 60°C with no significant population change within 1 h, in comparison to a 3-log reduction in the number of non-desiccated cells under identical conditions. A previous study also found that *Salmonella* cells that had previously adapted to desiccation conditions survived substantially longer in aged chicken litter than non-adapted control cells exposed to the same treatment (13).

However, as environmental conditions change, *Salmonella* must be able to rapidly adapt through alterations in gene expression in order to overcome stress efficiently. For example, this can be accomplished through attachment, which results in a phenotypic change allowing *Salmonella* to become more resistant to thermal stress than cells in planktonic form (167–170). Multiple studies have concluded that modifications of the membrane fatty acid composition of *Salmonella* strains were directly associated with their ability to resist thermal treatment where those cells with less membrane fluidity possessed greater thermal resistance (171–174). Similarly, in *E. coli*, the increase in membrane fluidity also leads to increased synthesis of HSPs, thus suggesting that membrane composition is directly related to thermal resistance (175). Under low temperatures, the physiological state of the cell can switch to a reversible, less fluid like lipid bilayer, whereas under high temperatures, the state of the cell switches to a membrane with higher fluidity. This is regulated by thermosensors (175). A review by O’Bryan et al. (12) noted that foodborne pathogens in contaminated food products possessing a high fat content demonstrated increased pathogenic potential.

A well-known gene encodes for nucleotide-associated protein (H-NS), a histone-like nucleotide-structuring protein, which has been associated with virulence factors as a temperature-dependent phenotype (65–70). This protein is considered a common transcriptional regulator that can be induced by thermal changes in *Salmonella*. At low temperature, H-NS binds to an AT-rich sequence and forms a complex. When temperature rises to 37°C (host body temperature), the binding capacity is reduced until dissociation occurs, leading to virulence gene expression. This mechanism was demonstrated in *E. coli* K-12 to control over 60% of the genes regulated by temperature including virulence factors (176). The association of H-NS with virulence has been verified in other pathogens such as *Salmonella* (69, 177–179), *Shigella, Yersinia enterocolitica*, and *Yersinia pseudotuberculosis* (180–182). Two studies were conducted to identify the mechanism of H-NS in *Salmonella*. The first study was performed on *S*. Typhimurium LT2, and it was noted that H-NS negatively regulated approximately 254 genes (69). The second study was carried out on *S*. Typhimurium 14028 (183), and it was discovered that 265 unique *Salmonella* genes were negatively associated with H-NS which contained low G + C content (183). In both studies, among the identified genes were those present in SPI-1, 2, 3, and 5 (184–186).

A more recent study by Pesingl et al. (187) demonstrated that protein-l-isoaspartyl methyltransferase (PIMT) is required by *Salmonella* to survive at 42°C and it in turn contributes to virulence capability in poultry (body temperature of 42°C). Proteins were susceptible to damage induced by thermal stress and thus PIMT assisted in prevention and repair of proteins. Under stress, aspartate is converted to iso-aspartate, which can lead to unfolded proteins and modified amino acids residues (188). Pesingl et al. (187) found that PIMT contributes to survival under both thermal and oxidative stress during stationary phase due to its direct role in protection of proteins at elevated temperatures. Therefore, further research is needed in the correlation between the heat shock responses and virulence gene expression and how their respective regulation patterns influence the pathogenic potential of *Salmonella*.

## Conclusions

*Salmonella* typically encounters various thermal stresses that can be host-specific and can represent a component of the overall immune and physiological response to infection. However, *Salmonella* spp. have developed thermal resistance mechanisms to overcome these changes in host temperature through the induction of stress response mechanisms. In particular, sigma factors play a leading role in thermal stress response. Preexposure to thermal stress can lead to an increase in pathogenic potential through activation and regulation of genes associated with thermal stress. This thermal stress response can influence the activation of genes associated with virulence and the general stress response allowing for *Salmonella* to overcome host defenses and establish infection. The type of host can also play a role on the ability to establish infection. A host with a higher body temperature than humans could activate thermal stress resistance mechanisms allowing for easier colonization and establishment of infection compared to a host with a body temperature at 37°C in which these thermal stress resistance mechanisms are not expressed. An understanding of the *Salmonella* thermal resistance is essential for elucidating survival and infection mechanisms. It could be useful to identify specific targets for prevention and treatment of *Salmonella* infections. Therefore, it is imperative that the proteins involved in regulation and activation of these genes be thoroughly studied in order develop novel strategies to reduce outbreak cases and infection in all types of hosts.

## Author Contributions

The contribution was equally distributed between all authors.

## Conflict of Interest Statement

The authors declare that the research was conducted in the absence of any commercial or financial relationships that could be construed as a potential conflict of interest.
